# Prenatal maternal PTSD as a risk factor for offspring ADHD: A register-based Swedish cohort study of 553 766 children and their mothers

**DOI:** 10.1192/j.eurpsy.2024.21

**Published:** 2024-03-01

**Authors:** Michael Borgert, Amandah Melin, Anna-Clara Hollander, Syed Rahman

**Affiliations:** 1 Epidemiology of Psychiatric Conditions, Substance Use and Social Environment (EPiCSS), Department of Global Public Health, Stockholm, Sweden; 2 Division of Insurance Medicine, Department of Clinical Neuroscience, Stockholm, Sweden

**Keywords:** attention deficit hyperactivity disorder, post-traumatic stress disorder, prenatal, risk factors

## Abstract

**Background:**

Attention-deficit hyperactivity disorder (ADHD) is highly heritable, though environmental factors also play a role. Prenatal maternal stress is suggested to be one such factor, including exposure to highly distressing events that could lead to post-traumatic stress disorder (PTSD). The aim of this study is to investigate whether prenatal maternal PTSD is associated with offspring ADHD.

**Method:**

A register-based retrospective cohort study linking 553 766 children born in Sweden during 2006–2010 with their biological parents. Exposure: Prenatal PTSD. Outcome: Offspring ADHD. Logistic regression determined odds ratios (ORs) with 95% confidence intervals (CIs) for ADHD in the offspring. Adjustments were made for potential covariates, including single parenthood and possible indicators of heredity measured as parental ADHD and maternal mental disorders other than PTSD. Subpopulations, excluding children with indicators of heredity, were investigated separately.

**Results:**

In the crude results, including all children, prenatal PTSD was associated with offspring ADHD (OR: 1.79, 95% CI: 1.37–2.34). In children with indicators of heredity, the likelihood was partly explained by it. Among children without indicators of heredity, PTSD was associated with offspring ADHD (OR: 2.32, 95% CI: 1.30–4.14), adjusted for confounders.

**Conclusions:**

Prenatal maternal PTSD is associated with offspring ADHD regardless of indicators of heredity, such as parental ADHD or maternal mental disorder other than PTSD. The association is partly explained by heredity and socioeconomic factors. If replicated in other populations, preferably using a sibling design, maternal PTSD could be identified as a risk factor for ADHD.

## Introduction

The prevalence of attention deficit hyperactivity disorder (ADHD) is different in children/adolescents and adults, both according to clinical and epidemiological studies [1]. However, according to a meta-analysis from 2012, 5.9% of youths meet the diagnostic criteria for ADHD [2]. According to another meta-analysis from 2009, the prevalence of this condition in adulthood is 2.5% [3]. Twin studies from high-income countries have found that genes and their interaction with the environment play a substantial role in causing ADHD – however, this varies over the life course [1, 4, 5], and establishing causality for specific environmental factors is challenging. Large-scale meta-analyses have found support for some risk factors, such as lead and phthalate metabolite burden, maternal use of acetaminophen and valproate during pregnancy, and specific air pollutants such as nitric oxide and organophosphate pesticides [1] but rejected other hypothesized risk factors such as sugar consumption, perfluoroalkyl substances via breast milk in infancy and maternal smoking during pregnancy [1]. However, whether childhood exposure to second-hand cigarette smoke is a risk factor for ADHD is less clear [1]. Pregnancy complications such as premature birth, low birth weight, and uterine hypoxia are also associated with ADHD development in the child [1]. Furthermore, low parental socioeconomic status is associated with offspring ADHD [1].

Post-traumatic stress disorder (PTSD) can develop after a very stressful or distressing event [6]. Estimates of PTSD prevalence differ between countries. A large study comparing data from 24 countries reported a lifetime PTSD prevalence in the general population ranging from 0.3 to 9.2%, with a mean lifetime PTSD prevalence of 3.2% [7]. A Swedish study estimated the incidence of specialized healthcare utilization for PTSD to be 0.7%, with a higher likelihood of utilization among women and migrants to Sweden [8]. Studies reporting the prevalence of PTSD in pregnant women are scarce, though one study estimates it to be 3.3% [9]. Important risk factors for PTSD development include female sex, chronic or major physical illness, substance use disorder, personal or family history of psychiatric disorders, adversity during childhood, cumulative exposure to potentially traumatic experiences, bereavement, and witnessing injury or death [6]. Importantly, PTSD is also a risk factor in and of itself, and a person diagnosed with PTSD has a higher likelihood of a number of somatic conditions, including cardiovascular disorders, type 2 diabetes, smoking [10], and substance use disorders [8, 11]. Thus, PTSD could both be a risk factor for and a consequence of substance use disorder (SUD) [8]. ADHD could also be a risk factor for PTSD among patients with SUD [12].

Studies have investigated the association between maternal trauma, such as losing a relative during pregnancy, and offspring ADHD [13]. Prenatal maternal stress has also been suggested as a possible risk factor for ADHD in offspring [14] and thus prenatal maternal PTSD (hereafter prenatal PTSD) may be a risk factor, although one systematic review underlines the low number of studies in this field, especially of those taking heritability into account [14]. To the best of our knowledge, this is the first study to investigate an association between prenatal PTSD and offspring ADHD.

In Sweden, all contact with health care professionals is recorded in local and national administrative registers that cover the entire population, creating a unique opportunity to conduct nationwide studies, including the total population. Our study aims to investigate the relationship between diagnosed prenatal PTSD and offspring childhood ADHD, and whether hereditary factors, measured as ADHD in parents and maternal mental illness, could explain offspring childhood ADHD. We also investigate whether substance use during pregnancy and other perinatal and socioeconomic factors impact this association. We hypothesize that prenatal PTSD is associated with offspring ADHD.

## Methods

### Study design and data sources

The study was a retrospective register-based cohort study. Data were extracted from a large, longitudinal database of linked national registers Psychiatry Sweden (PS) (https://ki.se/en/gph/psychiatry-sweden-the-register-linkage-epicss-group). This includes data on all people officially resident in Sweden after 1 January 1932, anonymized by Statistics Sweden (SCB) for research purposes. We obtained outcome, exposure, and covariate data from the following registers:

From Statistics Sweden (SCB):

- Total population register together with the medical birth register (see below) to identify cohort participants and obtain demographic data, for example, birth date, sex, and birth country.
- Multi-generation register to link participants to their parents.
- Longitudinal integrated database for health insurance and labor market studies (LISA) used for sociodemographic data, including disposable income.

From the National Board of Health and Welfare:

- National patient register (NPR) for psychiatric and somatic diagnoses. NPR includes data from inpatient care (available since 1973) and specialized outpatient care (available since 2001). However, the data on psychiatric outpatient care have had sufficient quality and coverage since 2006.
- Prescribed drug register for data on prescribed and purchased ADHD-related drugs since July 2005.
- Medical birth register for pregnancy/birth-related variables such as reported smoking during pregnancy and pregnancy/birth complications.

### Study population

The study population included all children born in Sweden during 2006–2010 who were still living in Sweden on 31 December 2016 (*n* = 554 513) and their biological parents. Due to limitations in data coverage, children born before 2006 were not included. Maternal data were not available for 747 children, and these children were therefore excluded. The final study population consisted of 553 766 children.

Due to the focus on factors other than hereditary behind ADHD, we created two subpopulations:

- children without parental ADHD (*n* = 535 651), and
- children with neither parental diagnosed ADHD nor diagnosed maternal mental disorders other than PTSD (*n* = 506 222).

### Exposure

The exposure variable was defined as diagnosed prenatal PTSD (International Classification of Diseases, ICD-10, F43.1), dichotomized to yes/no, where “yes” represented mothers who had received a PTSD diagnosis during the year of delivery or during the 2 years prior to delivery. The validity of the PTSD diagnoses used in this study is found to be high [15].

### Outcome

The outcome offspring ADHD was identified in NPR by the ICD-10 code F90 and/or the use of ADHD medication (ATC codes: N06BA01, N06BA02, N06BA04, N06BA07, and N06BA09). The outcome variable was dichotomized as yes/no.

### Covariates

Potential covariates were chosen based on ADHD risk factors which prior studies have found support for.

#### Offspring birth year

The cohort was divided according to the year of birth of the child (2006–2010).

#### Offspring sex assigned at birth

Female or male.

#### Maternal age (in years)

In four groups: <19, 19–35, >35, missing.

#### Paternal age (in years)

In four groups: <19, 19–35, >35, missing.

#### Parental ADHD

Dichotomized and defined in the same way as for offspring (see Section “Outcome”):

- Maternal ADHD
- Paternal ADHD
- Maternal and/or paternal ADHD

#### Maternal mental disorders except PTSD

Dichotomized

- Anxiety disorders except PTSD (ICD-10: F40-F43 except F43.1)
- Depressive disorders (ICD-10: F32-33)
- Nonaffective psychotic disorder (ICD-10: F20-29)
- Maternal mental disorder except PTSD combined (ICD-10: F40-F43 except F43.1, F32-33, F20-29)

#### Maternal smoking during pregnancy

Self-reported smoking of at least one cigarette a day at the time of the first antenatal visit dichotomized.

#### Maternal substance use

Dichotomized

- Alcohol use disorder (ICD-10: F10, G31.2, G62.1, G72.1, I42.6, K29.2, K70, K86.0, O35.4, P04.3, Q86.0, T51.0, X45, Y91, Z50.2, Z71.4)
- Other substance use disorders (ICD-10: F11-F19 except F17)
- Substance use combined

#### Maternal major somatic disorder

Dichotomized

- Diabetes mellitus (ICD-10: E10-E14)
- Respiratory disorder (ICD-10: J00-J99)
- Circulatory disorder (ICD-10: I00-I99 except I42.6)
- Maternal major somatic disorders combined

#### *Pregnancy/birth complication*s

Dichotomized

- Low birth weight (ICD-10: P07, O14-O15)
- Low APGAR score (0–6 after 5 min)
- Gestational hypertension (ICD-10: O14-O15)
- Pregnancy/birth complications combined

#### Migrant status

- Maternal birth country: Dichotomized as the mother being born in Sweden or not
- Paternal birth country: Dichotomized as the father being born in Sweden or not

#### Parity

Three groups: 1st child, 2nd, or later order, missing.

#### Disposable family income

According to Statistics Sweden, this variable was defined as annual disposable income based on total family income from all registered sources, including wages, welfare benefits, other social subsidies, and pensions. Individual disposable family income was derived by weighting total family income according to household size and composition, with younger children given lower weights than older household members. Divided into quintiles. The lowest quintile represented the lowest amount of disposable family income.

### Statistical analysis

Descriptive statistics for all variables are reported in Table 1. Exposed and unexposed groups were compared using Chi-square test (results presented in Appendix Table A). We fitted the logistic regression models to estimate odds ratios (ORs) with 95% confidence intervals (CIs) for offspring ADHD (Table 2) for the total population and for the two subgroups “children without parental ADHD” and “children with neither parental diagnosed ADHD nor diagnosed maternal mental disorders other than PTSD.” A range of potential covariates included in the register linkage were chosen as potential confounders, based on risk factors of ADHD, which prior studies have found support for. The initial Crude model included exposure and outcome only. Model I included the covariates decided a priori: child’s sex, year of birth, maternal age, paternal age, family situation, disposable family income, and parental country of birth. Model II included all variables in model I and maternal smoking during pregnancy, parity, pregnancy complications, and major somatic disorders. For the total population and the subgroup “children without parental ADHD,” we ran a modified version of model III, with child’s sex, ADHD in parents (only in the total population), and maternal mental disorders to see the effect of ADHD in parents without the other confounders. For the total population, we ran model IV, including all the factors in models I–III. All statistical analyses were done with the software IBM SPSS Statistics version 26.

**Table 1.** Descriptive statistics for children born in Sweden during 2006–2010

	Total population		Exposed group (children with prenatal PTSD)	
	Total 553 766 (100)		1 224 (0.2)	
*n* (%)	With ADHD	Without ADHD	With ADHD	Without ADHD
In total	14 719 (2.7)	539 047 (97.3)	57 (4.7)	1 167 (95.3)
Parental ADHD (comb.)	2 774 (18.8)	15 341(2.8)	22 (38.6)	128 (11.0)
Maternal mental disorder (comb.)	1 682 (11.4)	27 747 (5.1)	38 (66.7)	738 (63.2)
Smoking	4 534 (30.8)	81 473 (15.1)	25 (43.9)	379 (32.5)
Maternal substance use (comb.)	472 (8.2)	4732 (0.9)	15 (26.3)	103 (8.8)
Maternal somatic disorder (comb.)	216 (1.5)	5 472 (1.0)	2 (3.5)	36 (3.1)
Pregnancy complication (comb.)	2 049 (13.9)	54 639 (10.1)	10 (17.5)	143 (12.3)
Parental birth country, not Sweden	3 573 (24.3)	163 577 (30.3)	16 (28.1)	654 (56.0)
Offspring is female	3 606 (24.5)	266 127 (49.4)	13 (22.8)	566 (48.5)
Birth year offspring				
2006	4 140 (28.1)	102 662 (19.0)	14 (24.6)	172 (14.7)
2007	3 510 (23.8)	104 690 (19.4)	9 (15.8)	202 (17.3)
2008	2 995 (20.3)	107 066 (19.9)	15 (26.3)	222 (19.0)
2009	2 330 (15.8)	110 183 (20.4)	10 (17.5)	261 (22.4)
2010	1 744 (11.8)	114 446 (21.2)	9 (15.8)	310 (26.6)
Disposable income^a^				
1st quintile (lowest)	4 716 (32.0)	131 897 (24.5)	26 (45.6)	628 (53.8)
2nd quintile	2 801 (19.0)	76 994 (14.3)	13 (22.8)	198 (17.0)
3rd quintile	2 981 (20.3)	104 571 (19.4)	12 (21.1)	158 (13.5)
4th quintile	2 584 (17.6)	124 230 (23.0)	3 (5.3)	111 (9.5)
5th quintile (highest)	1 600 (10.9)	99 446 (18.4)	3 (5.3)	68 (5.8)

a No data available on disposable family income for *n* = 1 946.

**Table 2.** Odds ratios (ORs) with 95% confidence interval (CI) for offspring ADHD among children by exposure to prenatal PTSD

Population	Crude	Model I	Model II	Model III	Model IV
Children without prenatal PTSD	Reference group	Reference group	Reference group	Reference group	Reference group
Total population of children with prenatal PTSD (*n* = 553 766)	1.79 (1.37–2.34)	1.62 (1.23–2.13)	1.43 (1.09–1.88)	0.85 (0.65–1.13)	0.86 (0.65–1.14)
Children with prenatal PTSD without parental ADHD (*n* = 535 651)	1.48 (1.06–2.08)	1.42 (1.01–2.00)	1.25 (0.89–1.76)	0.87 (0.62–1.22)	0.92 (0.65–1.29)
Children with prenatal PTSD without parental ADHD or maternal psychiatric diagnosis other than PTSD (*n* = 506 222)	2.72 (1.56–4.76)	2.51 (1.42–4.45)	2.32 (1.30–4.14)^a^	NA	NA

*Note:* Model I – Including child’s sex, year of birth, maternal age, paternal age, family situation, disposable family income, and parental country of birth.

Model II – Model I + *Maternal smoking during pregnancy*, parity and pregnancy complications and major somatic disorders.

Model III – Including child’s sex, ADHD in parents (only in the total population) and maternal mental disorder.

Model IV – Including all the factors in models I–III.

a As this group is without ADHD in parents or any maternal mental disorder, model II is the mutually inclusive model for this group.

## Results

Table 1 shows the distribution of variables for the total population, and among the exposed and unexposed. Among children exposed to prenatal PTSD, more individuals were diagnosed with ADHD than among the unexposed (4.7 and 2.7%, respectively). The prevalence of ADHD was almost six times higher among mothers with PTSD than among those without (9.4% vs 1.6%). In mothers with PTSD, it was 12 times more common to have any other mental disorder compared to mothers without PTSD (63.4% vs 5.2%). Three-quarters of children diagnosed with ADHD were male. All investigated risk factors, except APGAR score and gestational hypertension, were significantly more prevalent in mothers with PTSD compared to their peers. For more detailed information about the prevalence of specific diagnoses included among maternal mental disorders, specific substances used by mothers, specific maternal somatic disorders, and specific pregnancy/birth complications, as well as for *p*-values of the differences in prevalence between the groups compared with the Chi-square test, please see Appendix Table A.

In the total population, children exposed to prenatal PTSD had a 79% (OR: 1.79, 95% CI: 1.37–2.34) higher likelihood of being diagnosed with ADHD in the crude model (Table 2). In model I, with child’s sex, year of birth, maternal age, paternal age, family situation, disposable family income, and parental country of birth, the association was attenuated but still significant (OR: 1.62, 95% CI: 1.23–2.13). However, in the fully expanded model, when including parental ADHD and maternal mental disorders, the association between prenatal PTSD and offspring ADHD was no longer significant.

In the subpopulation “children without parental ADHD”, the exposed had 42% higher odds of being diagnosed with ADHD (OR: 1.42, 95% CI: 1.01–2.00) compared to the unexposed, when the model included child’s sex, year of birth, maternal age, paternal age, family situation, disposable family income, and parental country of birth (Table 2). However, this significant association disappeared when including maternal mental health in the model.

Table 2 shows that, in the subpopulation “children without parental ADHD or maternal psychiatric diagnosis other than PTSD”, there was an association between prenatal PTSD and offspring childhood ADHD in the crude model (OR: 2.72, 95% CI:1.56–4.76) and in the model including child’s sex, year of birth, maternal age, paternal age, family situation, disposable family income, parental country of birth, maternal smoking, parity, pregnancy/birth complications and major somatic disorders (OR: 2.32, 95% CI:1.30–4.14).

For the association between parental PTSD and offspring ADHD in the subpopulation “children without parental ADHD or maternal psychiatric diagnosis other than PTSD”, individual co-variates such as child’s sex, year of birth, maternal age, paternal age, family situation, disposable family income, parental country of birth, smoking, parity, pregnancy/birth complications, and major somatic illness were included in the final model (see Table 3). When disposable family income was compared to the highest quintile, for each quintile, the lower the income, the higher the likelihood of offspring ADHD. Single parenthood among mothers with PTSD was associated with twice the likelihood for subsequent offspring ADHD (OR: 2.04, 95% CI: 1.80–2.32) compared to cohabiting parents. Smoking during pregnancy/birth was also associated with a higher likelihood of offspring ADHD (OR: 1.92, 95% CI: 1.83–2.02). Having at least one parent born abroad was associated with a significantly lower likelihood of offspring ADHD (OR: 0.81, 95% CI: 0.77–0.86). If the child was female, the likelihood was also significantly lower (OR: 0.30, 95% CI: 0.29–0.32).

**Table 3.** Odds ratios (ORs) with 95% confidence interval (CI) for offspring ADHD among children without parental ADHD or maternal psychiatric diagnosis other than PTSD for each variable in model II for offspring

Variable	Parameter	OR (95% CI)
Children without parental ADHD	Without maternal mental disorder	Reference group
	Without maternal mental disorder other than PTSD	2.32 (1.30–4.14)
Sex	Boy	Reference group
	Girl	0.30 (0.29–0.32)
Birth year offspring	2006	Reference group
	2007	0.80 (0.75–0.84)
	2008	0.64 (0.61–0.68)
	2009	0.46 (0.43–0.49)
	2010	0.32 (0.30–0.34)
Maternal age	<19	1.14 (0.94–1.38)
	19–35	Reference group
	>35	0.87 (0.82–0.93)
	Missing	–
Paternal age	<19	0.76 (0.51–1.12)
	19–35	Reference group
	>35	1.05 (1.00–1.10)
	Missing	0.87 (0.73–1.03)
Family situation	Cohabiting	Reference group
	Single parent	2.04 (1.80–2.32)
	Other family situation	1.61 (1.47–1.77)
	Missing	1.42 (1.30–1.55)
Disposable income	1st quintile (lowest)	2.14 (1.99–2.31)
	2nd quintile	1.99 (1.85–2.15)
	3rd quintile	1.71 (1.59–1.84)
	4th quintile	1.25 (1.16–1.34)
	5th quintile (highest)	Reference group
	Missing data	1.76 (1.20–2.60)
Migration status	Both parents born in Sweden	Reference group
	One parent born in Sweden	0.81 (0.77–0.86)
	Both parents born outside Sweden	0.54 (0.50–0.58)
Smoking	Non-smoking during pregnancy	Reference group
	Smoking during pregnancy	1.92 (1.83–2.02)
Parity	First child	Reference group
	2nd child or later order	0.75 (0.71–0.78)
	Missing	0.58 (0.50–0.68)
Pregnancy complications	No	Reference group
	Yes	1.16 (1.11–1.21)
Major somatic disorders	Yes	Reference group
	No	0.78 (0.64–0.94)

## Discussion

Our study aimed to investigate the relationship between diagnosed prenatal PTSD and offspring ADHD in childhood, and if this could be explained by hereditary factors, adjusted for known risk factors for ADHD such as perinatal pregnancy-related factors and socioeconomic factors [1]. As hypothesized, an association between maternal prenatal PTSD and childhood ADHD in offspring was found. Hereditary factors, measured as parental ADHD and maternal mental disorders other than PTSD, seem to be the major contributors to such associations in the total study population. Nonetheless, in the subpopulation “children without parental ADHD or maternal psychiatric diagnosis other than PTSD,” the likelihood of childhood ADHD was doubled among those with prenatal PSTD even after controlling for a range of strong confounders such as single parenthood and low disposable income [1].

Several studies have investigated the association between maternal stress and offspring ADHD [14], but not prenatal PTSD and offspring ADHD. To the best of our knowledge, this is the first study to examine whether PTSD in the years immediately before/during pregnancy could be a risk factor for ADHD in offspring, while also taking the influence of hereditary factors into account. If replicated, this means additional support for the hypothesis that stress is one of the environmental risk factors for ADHD. There are several theories as to why prenatal stress or PTSD could be associated with offspring ADHD. One such theory predicts that immune activation in women with stress is associated with neonatal functional brain connectivity and offspring behavior [14]. Another, the fetal programming hypothesis, speculates that since the fetus adapts to the prenatal environment, for example, through epigenetic changes, prenatal stress could generate lasting offspring adaptations such as shorter attention spans [16, 17]. Stress-related hormones could also play a part in this association [18]. It is speculated that, with more anxiety, the placenta becomes more permeable to cortisol and thus increases fetal exposure [18]. Low birth weight has been associated with ADHD [19, 20], and there is evidence supporting a link between prenatal maternal stress and low birth weight [21-23].

Distributions of several characteristics were significantly different between mothers with and without prenatal PTSD. Diagnosed ADHD, smoking during pregnancy, substance use, mental disorders, low disposable income, major somatic disorders, and being born outside of Sweden were more commonly observed in mothers with PTSD than in mothers without. This indicates that PTSD rarely is an isolated condition in otherwise healthy people, which makes it difficult to study PTSD alone. ADHD heredity (diagnosed ADHD in parents) confounded the crude association between prenatal PTSD and offspring ADHD. Considering the fact that about 50% of adults with ADHD have a comorbid mental disorder [24], it is likely that the association between prenatal maternal mental disorder and offspring ADHD is partly explained by shared heredity.

In the subgroup analysis of children without diagnosed ADHD in parents or maternal mental illness, prenatal PTSD also doubled the likelihood of childhood ADHD. In addition, several confounders doubled the likelihood of ADHD in the child, including single parenthood and low disposable income. The reason that single parenthood doubles the likelihood of offspring ADHD is unknown, but may be related to the fact that low disposable family income is known to be more common among single parents, and an association between parental low disposable income and offspring ADHD is found in previous studies [25, 26]. In addition, we found that reported smoking during pregnancy is associated with offspring ADHD. However, sibling- and twin-designed studies have shown that the association between smoking during pregnancy and ADHD is likely due to confounding because smoking during pregnancy is associated with ADHD/externalizing problems in the mother [25]. We therefore conclude that smoking during pregnancy is a confounding factor in our study as well.

## Strengths and limitations

An important strength of this study is the use of comprehensive register linkage, making it possible to study large numbers of participants over time. Using health registers minimizes the risk of recall bias, the PTSD diagnoses did not rely on mothers remembering whether they had PTSD during pregnancy. Furthermore, the large number of participants makes it more probable that true associations are found even if some misclassification occurs. Very few participants were excluded due to missing data. Another advantage of using record linkage is that the data includes many potential confounders in offspring and parents.

Although the sample was large, the number of mothers with prenatal PTSD who had offspring diagnosed with ADHD was only 57. Also, the multivariate logistical regression models included a range of covariates – thus lowering statistical power, possibly in turn increasing the risk of type II errors.

An important limitation is that we did not test for an interaction between genetic and environmental factors in the children. Neither did we have the opportunity to consider the severity of prenatal PTSD. Both factors are likely to introduce residual confounding. Sibling design/twin studies could constitute a better approach because this would include common heredity and many shared environmental factors.

This study relies on the health care system to have diagnosed both PTSD and ADHD accurately. This could be a limitation, as it might lead to underestimation of ADHD in both children and parents (as a confounder), as well as of PTSD in parents. With regard to the underestimation of ADHD in children in our study, the vast majority of the children diagnosed with ADHD were boys. It has been suggested that ADHD is underdiagnosed in girls. If this is the case in the study population, that would indicate an ascertainment bias, so that girls with ADHD are classified as not having ADHD. If this is the case, that could also lead to an underestimation of the total association.

PTSD is likely to be an underdiagnosed condition in psychiatry [8]. In our study, prenatal PTSD prevalence was 0.2%. This is a very low figure when compared to previous research that has found prevalence in prenatal mothers worldwide to be around 3% [7] but the same as the incidence of specialized healthcare utilization for PTSD in Sweden [8]. Studies finding higher prevalence often used data from prenatal PTSD screenings and not register data of specialized health care utilization for PTSD [8]. Register-based data only include cases so severe that the patient seeks care; hence, milder cases may not be included. This discrepancy might also suggest underreporting of PTSD in the present study. Thus, if there is an association, as the study implies, this might only be present among mothers with severe forms of PTSD. If a higher proportion of mothers with prenatal PTSD were identified, the association between prenatal PTSD and offspring ADHD could be stronger. However, it is also possible that the association would be weaker because those mothers who are classified as having PTSD in the current study are those with the most serious form of the disorder.

The children in our study were 6 to 10 years of age, and this is a limitation because the study did not include cases of ADHD diagnosed at an older age. Although this age range corresponds to the age when many boys are diagnosed with ADHD, girls and children with less hyperactivity are often diagnosed later [27]. This limitation leads to a possible underestimation of ADHD, and this could weaken the association found. ADHD was for a long time mainly diagnosed in children, and only later became more commonly diagnosed among adults [1]. Thus, ADHD diagnoses among parents are probably underestimated. However, the lower prevalence in adults might also be due to the fact that only about one in six youths with ADHD still meet the full diagnostic criteria for ADHD at age 25 [1]. Still, this could lead to the consequence that, in the subpopulation of children without parental ADHD, there are still children whose parents do have ADHD. This is most likely among persons with less severe symptoms because they are the least likely to be detected. As ADHD often is comorbid with other mental disorders, we tried to handle this likely underestimation of ADHD among the parents by excluding mothers with a mental disorder, that is, having a subpopulation of children with neither parental diagnosed ADHD nor diagnosed maternal mental disorders other than PTSD. This subpopulation probably has a lower underestimation of ADHD.

Despite PTSD being more common among migrants and, in particular, refugees [8] as compared with the Swedish-born majority population, it could still be under-detected among migrants [8]. Studies also show that ADHD is under-detected among children with a migrant background [28]. The under-detection of PTSD among migrant mothers and ADHD among migrant children could weaken the possible association in the total population.

The association between parental PTSD and offspring ADHD could be impacted by SUD. SUD is a risk factor for PTSD, and PTSD could also be a risk factor for SUD. Since substance use during pregnancy is a risk factor for offspring ADHD [27], there is a possibility that any association we have found is confounded by SUD. We have thus tried to include SUD in the models – however, doing so did not alter the association significantly, possibly due to low PTSD prevalence. Further research needs to find additional ways to eliminate this bias.

## Implications

If the finding that maternal PTSD is a risk factor for ADHD is replicated in other populations, this could lead to new insights in the etiology of ADHD, in understanding the risk factors for ADHD, and in developing possible strategies to prevent ADHD. Further studies should focus on the elimination of unmeasured confounding, possibly using a sibling design.

## Conclusion

Prenatal PTSD was associated with offspring ADHD. In children with exposure to parental ADHD or maternal mental disorders, ADHD was better explained by these hereditary factors. In children without parental ADHD or maternal mental disorders, there was still an association between prenatal PTSD and offspring ADHD, even after adjustment for a range of factors such as low disposable income. If replicated in other populations where sibling comparison design is possible, maternal PTSD could be more conclusively identified as a risk factor for offspring ADHD.

## Supporting information

Borgert et al. supplementary material Borgert et al. supplementary material

## Data Availability

Data cannot be shared publicly under the terms and conditions of ethical approval for Psychiatry Sweden.

## Supplementary material

The supplementary material for this article can be found at http://doi.org/10.1192/j.eurpsy.2024.21.
