# Supplementary material for: Prenatal maternal PTSD as a risk factor for offspring ADHD: A register-based Swedish cohort study of 553 766 children and their mothers
Source: Eur Psychiatry. 2024 Mar 1;67(1):e22. doi: 10.1192/j.eurpsy.2024.21 (PMC10966610; doi:10.1192/j.eurpsy.2024.21)
Supplement: Borgert et al. supplementary material [file S092493382400021Xsup001.docx]

**Table A**
*Participants: Descriptive statistics for children born in Sweden during 2006-2010 (n=553 766), including data associated with their biological parents*

|  | **Total (n=553 766)** | | | | **Prenatal PTSD (n=1 224; 0.2%)** | | | | **No prenatal PTSD (n=552 542; 99.8%)** | | | | **χ2** |
| --- | --- | --- | --- | --- | --- | --- | --- | --- | --- | --- | --- | --- | --- |
| n (%) | Offspring ADHD n=14 719; (2.7%) | | No Offspring ADHD n=539 047; (97.3%) | | Offspring ADHD n=57; (4.7%) | | No Offspring ADHD n=1 167; (95.3%) | | Offspring ADHD n=14 662; (2.7%) | | No Offspring ADHD n=537 880; (97.3 %) | |  |
| Parental ADHD (comb.) | 2 774 | (18.8) | 15 341 | (2.8) | 22 | (38.6) | 128 | (11.0) | 2 752 | (18.8) | 15 213 | (2.8) | < .001 |
| Maternal ADHD | 1 616 | (11.0) | 7 520 | (1.4) | 16 | (28.1) | 99 | (8.5) | 1 600 | (10.9) | 7 421 | (1.3) | < .001 |
| Paternal ADHD | 1 460 | (9.9) | 8 697 | (1.6) | 9 | (15.8) | 41 | (3.5) | 1 451 | (9.9) | 8 656 | (1.6) | < .001 |
| Maternal mental disorder (comb.) | 1 682 | (11.4) | 27 747 | (5.1) | 38 | (66.7) | 738 | (63.2) | 1 644 | (11.2) | 27 009 | (5.0) | < .001 |
| Anxiety disorder other than PTSD | 1 182 | (8.0) | 19 383 | (3.6) | 35 | (61.4) | 528 | (45.2) | 1 147 | (7.8) | 18 855 | (3.5) | < .001 |
| Depressive disorder | 880 | (6.0) | 13 856 | (2.6) | 18 | (31.6) | 477 | (40.9) | 862 | (5.9) | 13 381 | (2.5) | < .001 |
| Non-affective psychosis | 66 | (0.4) | 900 | (0.2) | 5 | (8.8) | 44 | (3.8) | 61 | (0.4) | 856 | (0.2) | < .001 |
| Smoking | 4 534 | (30.8) | 81 473 | (15.1) | 25 | (43.9) | 379 | (32.5) | 4 509 | (30.8) | 81 094 | (15.1) | < .001 |
| Alcohol use disorder | 260 | (1.8) | 2 771 | (0.5) | 8 | (14.0) | 39 | (3.3) | 252 | (1.7) | 2 732 | (0.5) | < .001 |
| Other substance use disorder | 212 | (1.4) | 1 961 | (0.4) | 7 | (12.3) | 64 | (5.5) | 205 | (1.4) | 1 897 | (0.4) | < .001 |
| Maternal substance use (comb.) | 472 | (8.2) | 4732 | (0.9) | 15 | (26.3) | 103 | (8.8) | 457 | (3.1) | 4629 | (0.9) | < .001 |
| Maternal somatic disorder (comb.) | 216 | (1.5) | 5 472 | (1.0) | 2 | (3.5) | 36 | (3.1) | 214 | (1.5) | 5 436 | (1.0) | < .001 |
| Diabetes mellitus | 30 | (0.2) | 641 | (0.1) | 1 | (1.8) | 7 | (0.6) | 29 | (0.2) | 634 | (0.1) | < .001 |
| Respiratory disorder | 122 | (0.8) | 3 098 | (0.6) | 0 | (0.0) | 23 | (2.0) | 122 | (0.8) | 3 075 | (0.6) | < .001 |
| Circulatory disorder | 79 | (0.5) | 1 894 | (0.4) | 1 | (1.8) | 8 | (0.7) | 78 | (0.5) | 1 886 | (0.4) | < .05 |
| Pregnancy complication (comb.)^1^ | 2 049 | (13.9) | 54 639 | (10.1) | 10 | (17.5) | 143 | (12.3) | 2 039 | (13.9) | 14 207 | (2.6) | < .05 |
| Low Birth Weight | 1 153 | (7.8) | 28 599 | (5.3) | 3 | (5.3) | 80 | (6.9) | 1 150 | (7.8) | 28 519 | (5.3) | < .05 |
| Low APGAR (0-6)^2^ | 203 | (1.4) | 6 007 | (1.1) | 2 | (3.5) | 19 | (1.6) | 201 | (1.4) | 5 988 | (1.1) | .056 |
| Gestational Hypertension | 970 | (6.6) | 26 702 | (5.0) | 6 | (10.5) | 68 | (5.8) | 564 | (3.8) | 26 634 | (5.0) | .092 |
| Parental born outside Sweden | 3 573 | (24.3) | 163 577 | (30.3) | 16 | (28.1) | 654 | (56.0) | 3 557 | (24.3) | 162 923 | (30.3) | < .001 |
| Mother born outside of Sweden | 2 217 | (15.1) | 122 835 | (22.8) | 11 | (19.3) | 572 | (49.0) | 2 206 | (15.0) | 122 263 | (22.7) | < .001 |
| Father born outside of Sweden | 2 845 | (19.3) | 128 459 | (23.8) | 13 | (22.8) | 569 | (48.8) | 2 832 | (19.3) | 127 890 | (23.8) | < .001 |
| Offspring is female | 3 606 | (24.5) | 266 127 | (49.4) | 13 | (22.8) | 566 | (48.5) | 3 593 | (24.5) | 265 561 | (49.4) | .325 |
| Offspring birth year |  |  |  |  |  |  |  |  |  |  |  |  | < .001 |
| 2006 | 4 140 | (28.1) | 102 662 | (19.0) | 14 | (24.6) | 172 | (14.7) | 4 126 | (28.1) | 102 490 | (19.1) |  |
| 2007 | 3 510 | (23.8) | 104 690 | (19.4) | 9 | (15.8) | 202 | (17.3) | 3 501 | (23.9) | 104 488 | (19.4) |  |
| 2008 | 2 995 | (20.3) | 107 066 | (19.9) | 15 | (26.3) | 222 | (19.0) | 2 980 | (20.3) | 106 844 | (19.9) |  |
| 2009 | 2 330 | (15.8) | 110 183 | (20.4) | 10 | (17.5) | 261 | (22.4) | 2 320 | (15.8) | 109 922 | (20.4) |  |
| 2010 | 1 744 | (11.8) | 114 446 | (21.2) | 9 | (15.8) | 310 | (26.6) | 1 735 | (11.8) | 114 136 | (21.2) |  |
| Disposable family income^3^ |  |  |  |  |  |  |  |  |  |  |  |  | < .001 |
| 1st quintile (lowest) | 4 716 | (32.0) | 131 897 | (24.5) | 26 | (45.6) | 628 | (53.8) | 4 690 | (32.0) | 131 269 | (24.4) |  |
| 2nd quintile | 2 801 | (19.0) | 76 994 | (14.3) | 13 | (22.8) | 198 | (17.0) | 2 788 | (19.0) | 76 796 | (14.3) |  |
| 3rd quintile | 2 981 | (20.3) | 104 571 | (19.4) | 12 | (21.1) | 158 | (13.5) | 2 969 | (20.2) | 104 413 | (19.4) |  |
| 4th quintile | 2 584 | (17.6) | 124 230 | (23.0) | 3 | (5.3) | 111 | (9.5) | 2 581 | (17.6) | 124 119 | (23.1) |  |
| 5th quintile (highest) | 1 600 | (10.9) | 99 446 | (18.4) | 3 | (5.3) | 68 | (5.8) | 1 597 | (10.9) | 99 378 | (18.5) |  |

*Note.* Within brackets are the percentages of individuals compared to the sum total of individuals mentioned in the column title. Chi-square is calculated to show significant differences in distribution in the groups Prenatal PTSD and No prenatal PTSD.

^1^ No data available on pregnancy complication for n=20 651.

^2^ No data available on low APGAR for n=21 866.

^3^ No data available on disposable family income for n=1 946.
